# Mycophenolic acid directly protects podocytes by preserving the actin cytoskeleton and increasing cell survival

**DOI:** 10.1038/s41598-023-31326-z

**Published:** 2023-03-15

**Authors:** Seif El Din Abo Zed, Agnes Hackl, Katrin Bohl, Lena Ebert, Emilia Kieckhöfer, Carsten Müller, Kerstin Becker, Gregor Fink, Kai-Dietrich Nüsken, Eva Nüsken, Roman-Ulrich Müller, Bernhard Schermer, Lutz T. Weber

**Affiliations:** 1 Faculty of Medicine and University Hospital Cologne, Department of Pediatrics, University of Cologne, Cologne, Germany; 2 Faculty of Medicine and University Hospital Cologne, Department II of Internal Medicine and Center for Molecular Medicine Cologne, University of Cologne, Cologne, Germany; 3 Faculty of Medicine and University Hospital Cologne, Pharmacology at the Laboratory Center, Department of Therapeutic Drug Monitoring DE, University of Cologne, Cologne, Germany; 4 Faculty of Medicine and University Hospital Cologne, Cologne Center for Genomics (CCG), University of Cologne, Cologne, Germany; 5 Cologne Cluster of Excellence on Cellular Stress Responses in Ageing-Associated Diseases (CECAD), University of Cologne, Cologne, Germany; 6 Faculty of Medicine and University Hospital Cologne, Center for Rare Kidney Diseases Cologne, University of Cologne, Cologne, Germany

**Keywords:** Paediatric kidney disease, Podocytes, Transcriptomics

## Abstract

Mycophenolate Mofetil (MMF) has an established role as a therapeutic agent in childhood nephrotic syndrome. While other immunosuppressants have been shown to positively affect podocytes, direct effects of MMF on podocytes remain largely unknown. The present study examines the effects of MMF’s active component Mycophenolic Acid (MPA) on the transcriptome of podocytes and investigates its biological significance. We performed transcriptomics in cultured murine podocytes exposed to MPA to generate hypotheses on podocyte-specific effects of MPA. Accordingly, we further analyzed biological MPA effects on actin cytoskeleton morphology after treatment with bovine serum albumin (BSA) by immunofluorescence staining, as well as on cell survival following exposure to TNF-α and cycloheximide by neutral red assay. MPA treatment significantly (adjusted *p* < 0.05) affected expression of 351 genes in podocytes. Gene Ontology term enrichment analysis particularly clustered terms related to actin and inflammation-related cell death. Indeed, quantification of the actin cytoskeleton of BSA treated podocytes revealed a significant increase of thickness and number of actin filaments after treatment with MPA. Further, MPA significantly reduced TNFα and cycloheximide induced cell death. MPA has a substantial effect on the transcriptome of podocytes in vitro, particularly including functional clusters related to non-immune cell dependent mechanisms. This may provide a molecular basis for direct beneficial effects of MPA on the structural integrity and survival of podocytes under pro-inflammatory conditions.

## Introduction

Mycophenolic acid (MPA) is the active component of the prodrug mycophenolate mofetil (MMF). MPA acts as a highly selective and potent inhibitor of the inosine-5′-monophosphate dehydrogenase (IMPDH), leading to a depletion of the pool of guanosine triphosphate (GTP) and deoxy GTP^1,2^. It has been shown that MPA binds the type II isoform of IMPDH, expressed in activated B- and T-lymphocytes, with an affinity five times higher than the constitutively expressed type I isoform, leading to an effective blockage of lymphocyte proliferation while having only minor effects on the multiplication of other cell types^3,4^. This mechanism of action, which results in a comparably low adverse risk profile, has led to a wide use of MMF in the field of organ transplantation, where it emerged as an effective drug for prevention of acute rejection of, inter alia, kidney allografts^5^. Further, MMF has been a vital part of systemic lupus erythematosus therapy, especially in patients with solid organ manifestations, such as lupus nephritis^6^. MMF is also recommended as a glucocorticoid sparing agent for steroid sensitive nephrotic syndrome in the recently published KDIGO 2021 Clinical Practice Guideline for the Management of Glomerular Diseases^7^. Its use in treating the most prevalent cause of glomerular disease in children, the idiopathic nephrotic syndrome (INS), has been constantly expanding^8^. Despite extensive research the pathomechanism of INS has not yet been fully elucidated. Traditionally, INS has been viewed as a T-lymphocyte derived disease, but successful treatment with the anti-CD20 antibody rituximab has amended this viewpoint^9–12^. Importantly, there has been increasing evidence for a central role of podocytes in the pathogenesis of INS. Podocytes are terminally differentiated cells, whose foot processes (FP), interdigitate with those of neighboring podocytes. This connection constitutes the outer layer of the glomerular filtration barrier, the slit diaphragm (SD)^13^. During disease the actin cytoskeleton undergoes dynamic changes, thereby inducing FP effacement, which leads to a reduced compressive force on the glomerular membrane and consequently, to the loss of albumin and other proteins into the urine^14–16^. Other immunosuppressants used in treating INS such as glucocorticoids, cyclosporine A and rituximab positively affect the actin cytoskeleton of podocytes as part of their antiproteinuric effect^17–19^. Research into the effect of MPA on kidney cells is scarce. In both in vitro and in vivo experiments, it has been shown that MPA inhibits the proliferation of mesangial cells, through the depletion of the guanosine pool^20,21^. With regard to podocytes, MPA showed a preservative effect on nephrin expression in adriamycin-induced nephritis and diabetes mellitus models^22,23^. In the latter model, MPA was able to reduce podocyte apoptosis through a reduction of Bax and cleaved caspase-3 (CC3) protein levels. Transcriptomic analysis of murine kidneys in a lupus nephritis model showed an increase in actin associated terms after treatment with MMF^24^. In the same study, MMF reduced the activation of Rac1 of cultured podocytes compared to controls. To improve our insight into the effect of MPA on podocytes, we were prompted to further investigate direct, non-immune cell mediated responses through which MPA could favorably affect these cells. We therefore performed a transcriptomic analysis of MPA treated podocytes, followed by an investigation of the functional impact of the discovered changes in mRNA levels.

## Methods

### Cell culture and MPA treatment

**Figure 1:**
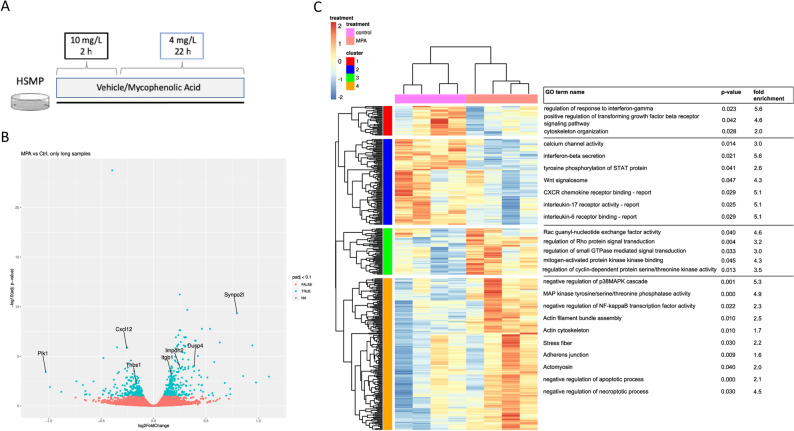
Treatment with MPA leads to changes in the transcriptome of podocytes. (**A**) Conditionally immortalized podocytes were differentiated treated with either Vehicle or Mycophenolic Acid (MPA) for 24 h and subjected to RNAseq analysis. (**B**) Volcano Plot visualizing the effects of MPA on the transcriptome of podocytes. Blue, adjusted *p*-value (padj) < 0.05; red, padj > 0.05. MPA treatment resulted in 130 significantly downregulated and 221 significantly upregulated genes. (**C**) Genes were sorted into a heatmap according to their z-score transformation value, resulting in four clusters. Cluster 1, red, downregulated genes. Cluster 2, blue, highly downregulated genes. Cluster 3, green, upregulated genes. Cluster 4, orange, highly upregulated genes. (**B**) Clusters were subjected to a Gene Ontology (GO)-term enrichment analysis. Important significantly enriched terms are summarized for each cluster.

A conditionally immortalized murine podocyte cell line (kindly provided by Dr. P. Mundel) was cultivated on type I collagen (A1064401—Invitrogen) coated culture dishes and kept in RPMI-1640 Medium + GlutaMAX (#61870036, Gibco), supplemented with 10% fetal bovine serum (FBS, 10270106—Gibco), 1% HEPES solution (H0887—Sigma) and 1% sodium pyruvate (S8636—Sigma) at 33 °C in the presence of recombinant mouse Interferon-$\gamma$(INF-$\gamma$, #315–05, PeproTech) to allow proliferation at permissive conditions. This was followed by incubation at 37 °C without additional INF-$\gamma$ for 10 days to induce differentiation at the non-permissive condition, as described previously^25^. Differentiation was confirmed by synaptopodin staining (data not shown). Cells were regularly checked for Mycoplasma infection. After differentiation, cells were treated with MPA (M3536—Sigma-Aldrich) with a concentration of 10 mg/L for 2 h, followed by a concentration of 4 mg/L for 22 h. MPA concentrations were chosen based on the pharmacokinetics of MPA plasma concentrations measured in children with idiopathic nephrotic syndrome^26^. Cells of the control group received the respective amount of methanol as vehicle (Fig. 1A).

### RNA extraction, cDNA library construction and sequencing

Cells were treated according to treatment protocol A (Fig. 1A). Total RNA was extracted using an RNA-Extraction Kit by Zymo Research (R2052direct-zol RNA-miniprep) according to the manufacturers’ instruction, mRNA sequencing was performed at the Cologne Center for Genomics. Libraries were prepared using the Illumina® Stranded TruSeq® RNA sample preparation Kit. Library preparation started with 2 µg total RNA. After poly-A selection (using poly-T oligo-attached magnetic beads), mRNA was purified and fragmented using divalent cations under elevated temperature. The RNA fragments underwent reverse transcription using random primers. This was followed by second strand cDNA synthesis with DNA Polymerase I and RNase H. After end repair and A-tailing, indexing adapters were ligated. The products were then purified and amplified (14 PCR cycles) to create the final cDNA libraries. After library validation and quantification (Agilent Tape Station), equimolar amounts of library were pooled. The pool was quantified by using the Peqlab KAPA Library Quantification Kit and the Applied Biosystems 7900HT Sequence Detection System. The pool was sequenced on an Illumina NovaSeq6000 sequencing instrument with a PE100 protocol^27,28^.

### RNAseq data mining (GO-term)

The reads were trimmed with Trimmomatic (version 0.36) using default parameters^29^. Additionally, up to ten low quality bases in the beginning of the reads were removed. The trimmed reads were mapped to the GRCm39 mouse reference genome with STAR version 2.6.0 using default parameters^30^. The analysis of differential gene expression was performed using the R package DeSeq2 (version 1.26.0)^31,32^. For visualization purposes the batch effect was removed using the method removeBatchEffect from the R package limma^33^. The enrichment analysis was done using the R package topGO^34,35^. For the heatmap and clustering the R package pheatmap was used^36^. The Z-transformed gene expression of genes with an adjusted *p*-value (padj) < 0.05 (calculated by DeSeq2) in the comparison MPA vs control was visualized and clustered in the heatmap.

### Immunofluorescence and quantification of the actin cytoskeleton

**Figure 2:**
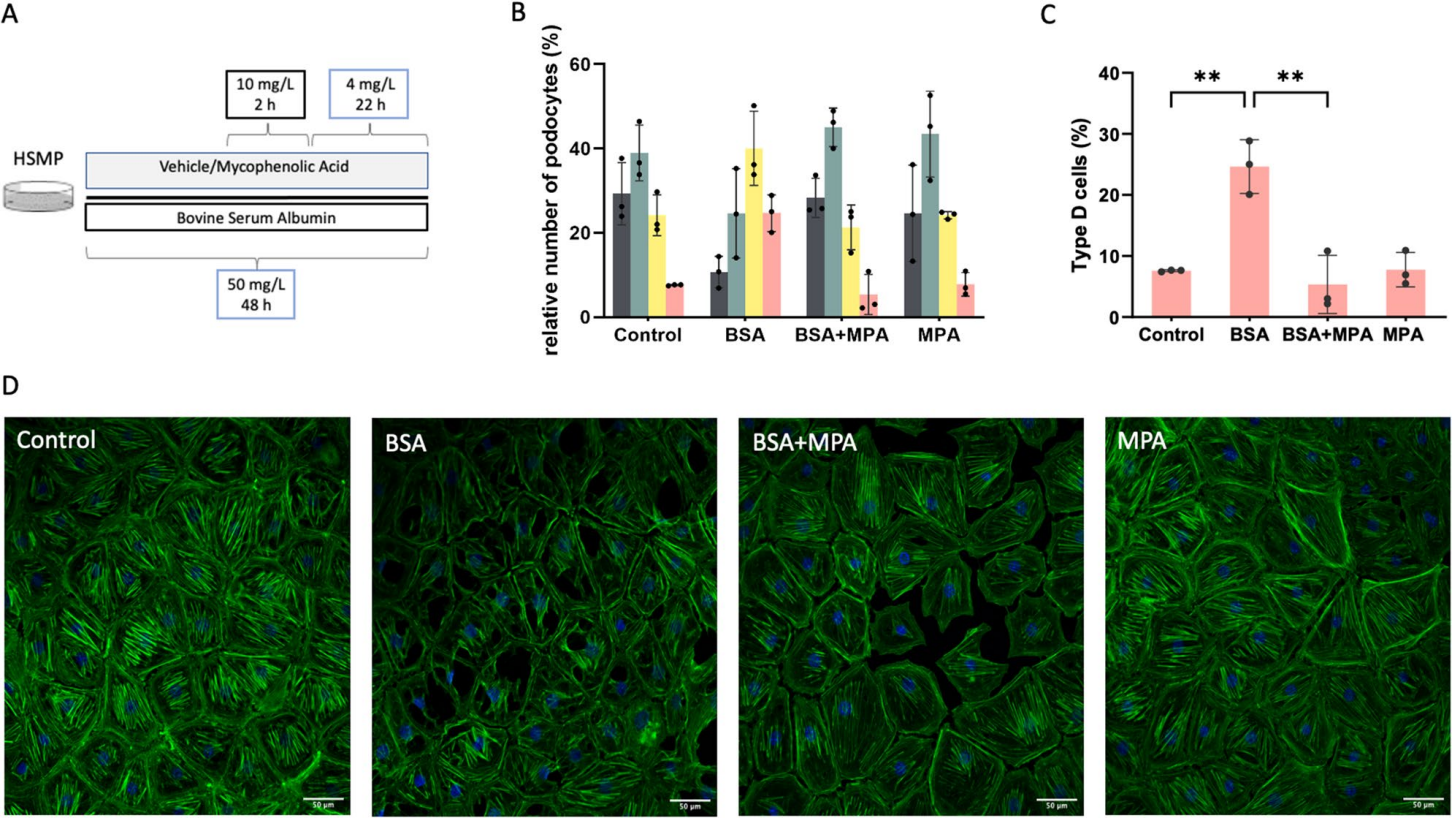
MPA protects the actin cytoskeleton from BSA induced injury. (**A**) Cells were treated with bovine serum albumin (BSA) for 48 h and received either MPA or Vehicle for the second 24 h. The actin cytoskeleton was visualized using immunofluorescence staining. (**B**) Quantification of different cell types after treatment with bovine serum albumin (BSA) and Mycophenolic Acid (MPA). Grey = Type A: > 90% of cell area filled with thick cables; green = type B: at least two thick cables running under the nucleus, with the rest of the cell area filled with fine cables; yellow = type C: no thick cables but some cables present; red = type D: no cables visible in the central area of the cell. BSA treatment decreases healthy Type A and B cells, while increasing less healthy Type C and D cells. Additional MPA treatment reverses this effect in all categories, increasing Type A and B cells compared to BSA only, while decreasing Type C and D cells. The treatment with only MPA resulted in a similar distribution of cell types as in controls. At least 90 cells were analyzed per sample. (**C**) Quantification of Type D cells for each treatment. Treatment with BSA significantly increases the number of cableless D cells. In contrast additional treatment with MPA significantly decreases the number of D cells compared to treatment with only BSA **p < 0.01. (**D**) Representative confocal images of the podocytes after each respective treatment. Green, phalloidin. Blue, DAPI.

Undifferentiated podocytes were seeded on coverslips and differentiated as described above. After differentiation, cells were incubated in medium with fatty-acid free, low endotoxin bovine serum albumin (BSA, A8806 –Sigma-Aldrich) at a concentration of 50 mg/ml for 48 h (BSA + vehicle [veh]). We used the study design of Yoshida et al.^37^ as orientation but adjusted the concentration of BSA to reach a robust injury model, where Type C and Type D cells prevail. The treatment group received additional MPA treatment in the second half of the 48 h (BSA + MPA). An additional group was treated only with MPA (MPA). Cells without BSA exposition and MPA treatment served as controls (Fig. 2A). The treated cells were then fixed with 4% paraformaldehyde prior to blocking and permeabilization with 5% Normal Donkey Serum and 0.1% Triton X-100 in phosphate buffered saline solution (PBS) for 30 min. Cells stained for synaptopodin were incubated with a synaptopodin antibody (S9442—Sigma-Aldrich, 1:500) overnight, followed by incubation with the appropriate secondary antibody at room temperature (RT) for 60 min (Alexa Fluor® 488 AffiniPure Donkey Anti-Rabbit IgG (H + L), 1:250). Cells stained for actin were incubated with fluorescently conjugated phalloidin at RT for 60 min (647P1-33—Dyomics, 1:50). The coverslips were then mounted with Prolong Gold and DAPI. Images were acquired with a Zeiss Meta 710 Confocal Laser Scanning Microscope and processed with ImageJ. For quantitative analysis of actin fiber assembly patterns, a previously described scoring system was adapted, and images were scored by an observer blinded to cell treatment^38^. At least 90 cells were analyzed per sample. The four groups describing the different actin fiber assembly patterns were defined as follows: Type A: 90% of cell area filled with thick cables; type B: at least two thick cables running under the nucleus, the rest of the cell area filled with fine cables; type C: no thick cables but some cables present; and type D: no cables visible in the central area of the cell (Fig. 2B).

### Measurement of cell viability

**Figure 3:**
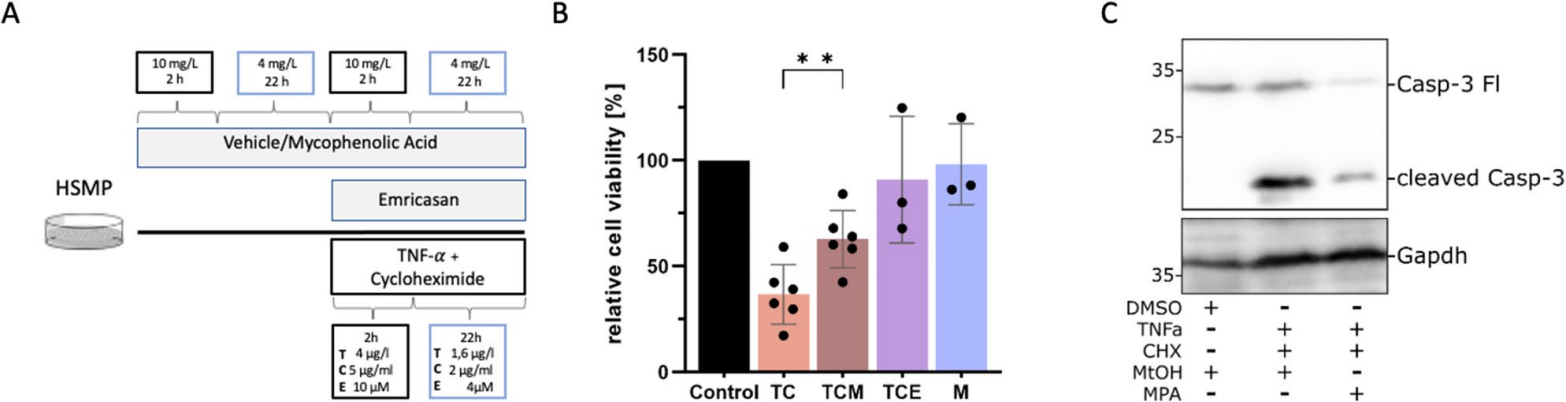
MPA increases cell viability during TNF-α and CHX induced cell death. (**A**) Cells were treated with either Vehicle or MPA (M) for 48 h, or Emricasan (E) for 24 h. All cells received Tumor Necrosis Factor-α (T) and Cycloheximide (C) for 24 h. An additional control group received only MPA for 48 h (M). This resulted in 5 treatment groups (Control, TC, TCM, TCE and M), which were analyzed with a cell viability assay. (**B**) Quantification of cell viability after treatment with combinations of Tumor Necrosis Factor-α (TNF-α; T), Cycloheximide (CHX; C), Mycophenolic Acid (MPA; M) and Emricasan (E). Control cells received only vehicles. Cell viability of each sample was normalized against the control of their respective passage. TC-treatment resulted in a drastic decrease of cell viability. Additional MPA treatment significantly increases cell viability compared to TC only (36.8 vs. 62.9%). Emricasan was able to prevent cell death in a very high degree (91%). Treatment with only MPA showed no signs for proliferative activity of the drug. **p < 0.01 (**C**) Western Blot Analysis of cleaved Caspase-3 levels. Lane 1, Control. Lane 2, TC. Lane 3, TCM. Treatment with TC results in a steep increase of cleaved Caspase-3 levels. TCM treated cells display a reduction of cleaved Caspase-3 compared to TC treated cells. Fl, full length. DMSO, Dimethyl sulfoxide. MtOH, Methanol. Gapdh, Glyceraldehyde 3-phosphate dehydrogenase. The displayed blot was cropped for better visualization. The boxes exemplify different regions of the same gel. The uncropped blots can be found as Supplementary Fig. 1.

Cells were seeded and differentiated in a 96-well culture dish. After differentiation, the treatment group was pretreated with MPA for 24 h. Afterwards cells were treated with recombinant mouse Tumor Necrosis Factor-α (TNF-α; Recombinant Mouse TNF-alpha [aa 80–235] Protein, #410-MT, R&D Systems, 20 ng/ml), Cycloheximide (CHX; C4859, Sigma-Aldrich, 5 µg/ml) and MPA for an additional 24 h (TCM). We used TNF-α in combination with the synergistically acting CHX in order to reach a high susceptibility to apoptosis^39,40^. Other treatment groups included cells receiving only TNF-α and CHX (TC), as well as cells receiving TNF-α, CHX and a pan-caspase inhibitor, Emricasan (SEL-S7775, Biozol) (TCE)^41^. Cells receiving only the vehicles of all respective agents served as control. An additional control group received only MPA for 48 h (M). The treatment schedules and concentrations of all agents are visualized in Fig. 3A. During the last 2 h of treatment, neutral red was added to each well. Next, cells were washed, and the dye was extracted out of viable cells with a destaining solution (50% EtOH, 49% VE H2O, 1% acetic acid). The absorbance of each well was measured through a spectrophotometer at 540 nm. The amount of viable cells was calculated as a ratio of the absorbance of each well to the absorbance of their respective control. Each condition was conducted with triplets of three different passages.

### Western blots related to cell viability

Cells of the treatment groups Control, TC and TCM were treated as described above (Fig. 3A). Treated cells were scraped off the dishes with ice cold PBS and cell pellets were isolated through centrifugation at 5.000 rpm for 5 min, before being snap frozen in liquid nitrogen. The cell pellets were resuspended in RIPA Buffer with additional Na3VO4 (20 µl/ml) and phenylmethylsulfonylfluoride (4,4 µl/ml) and then subject to the BCA Method to determine the amount of total protein. 15 µg of total protein were loaded per lane on 10% or 12% SDS-Page gels. The separated proteins were transferred onto polyvinylidene difluoride membranes, which were blocked in 5% BSA for 1 h. Next, the membranes were probed with primary antibodies overnight at 4 °C. After washing, the membranes were incubated with the secondary antibody at RT for 1 h. The signals were visualized with SuperSignal West Femto Maximum Sensitivity Substrate (#34095, Thermo Scientific) and bands were quantified using Fiji-ImageJ Version 2.1.0/1.53c^42^. Protein expression was quantified as the ratio of a specific band to glyceraldehyde-3-phosphate dehydrogenase (GAPDH).

The following primary antibodies were used in this study: cleaved Caspase-3 (Asp175) Antibody #9661 (Cell Signaling Technology), 1:1.000; GAPDH (D16H11) XP® Rabbit mAb#5174 (Cell Signaling Technology), 1:1.000. A horseradish peroxidase-conjugated goat antibody against rabbit (Cy™3 AffiniPure Donkey Anti-Rabbit IgG (H + L), AB_2307443, Jackson Immuno Research) was used as a secondary antibody (1:30.000).

### Statistical analysis

A two-way ANOVA test was used to compare the quantification of the actin cytoskeleton. A t-test with Welch’s correction was used to compare the results of the cell viability assay. Results with *p* < 0.05 were considered statistically significant. Results are reported as mean $\pm$ SEM.

## Results

### RNA sequencing reveals a significant direct effect of MPA on the transcriptome of podocytes

To gain a profound overview about the MPA-induced processes, we conducted an RNA sequencing (RNAseq) of MPA-treated podocytes. The experimental design is shown in Fig. 1A. We identified 351 genes that met the criterion of an adjusted *p* < 0.05 in order to be considered significantly affected by MPA treatment compared to controls.

A volcano plot visualizes the distribution of gene transcripts, showing that of the 351 differentially expressed genes 130 were significantly reduced and 221 were significantly increased (Fig. 1B). Both isoforms of *IMPDH* were highly expressed in all samples, with a consistently higher read count for *IMPDH II* (Suppl. Data). Interestingly, *IMPDH II* showed a significant, compensatory upregulation upon MPA treatment (log2FC 0.26, padj = 0.0002).

In order to better reveal inherent relations between these genes, we organized our data by the z-score transformed gene expression of each gene (Fig. 1C). Comparison of the data demonstrates a considerable consistency of expression changes inside each condition. Four gene meta-clusters were identified: down-regulated (Cluster 1), highly down-regulated (Cluster 2), up-regulated (Cluster 3) and highly up-regulated (Cluster 4).

To clarify molecular functions and biological processes affected by MPA-regulated genes and to address their relevance in podocytes, we next performed enrichment analysis for each cluster using Gene Ontology (GO). Figure 1C also depicts a selection of significant terms associated with each cluster. Complete result tables of enrichment are provided as supplementary information (Supplementary Dataset File 1–4). Looking at the results of the enrichment analysis we could outline two major groups of terms that were of particular interest.

### MPA treatment increases actin associated terms and genes

Looking at the significant terms of the clusters with genes induced upon MPA treatment (cluster 3 and cluster 4), we noticed an accumulation of terms related to the cytoskeletal structure of the cell. On the one hand, we had an increase of terms narrowing down the cellular components affected by the differentially expressed genes, like *actin cytoskeleton* (fold enrichment [FE] 1.7, *p* = 0.01), *stress fiber* (FE 2.2, *p* = 0.03), or *actomyosin* (FE 2.0, *p* = 0.04) (Fig. 1C). On the other hand, we could also see an increase in terms associated with the dynamic changes of those cellular components, like *actin filament bundle assembly* (FE 2.5, *p* = 0.01) and *regulation of Rho protein-* and *small GTPase mediated signal transduction* (FE 3.2 and 3.0, *p* = 0.004 and 0.03 respectively). Genes annotated to the terms of this subgroup include *SYNPO2L* (log2FC 0.80, padj = 466E-10), an actin associated protein from the synaptopodin family, and *ITGB1* (log2FC 0.17, padj = 0.0006), a protein crucial for cytoskeletal organization and signal transduction in podocytes. Further, we could see an upregulation of guanine nucleotide exchange factors (e.g., *ARHGEF2*), downstream effectors of RhoA (e.g., *ROCK2*) and other proteins that influence the activity of smallGTPases (e.g., *AMOT*) (Table 1).

**Table 1:** Genes associated to the actin cytoskeleton.

Name	Protein name	Adjusted *p*-value	Log2-fold change
SYNPO2L	Synaptopodin-2-like protein	4.66E-10	0.80
DRR1	Actin-associatedprotein FAM107A	0.0243	0.39
TXNRD1	Thioredoxin reductase 1	2.38E-08	0.25
MYL12B	Myosin regulatory light chain 12B	8.11E-07	0.23
CD157	ADP-ribosyl cyclase/cyclic ADP-ribose hydrolase 2	1.03E-06	0.22
ALDOA	Fructose-bisphosphatealdolase A	1.21E-07	0.21
ITGB1	Integrin beta-1	0.0006	0.17
ARHGEF9	Rho guanine nucleotide exchange factor 9	0.0086	0.31
ARHGEF2	Rho guanine nucleotide exchange factor 2	0.0164	0.15
ROCK2	Rho-associatedproteinkinase 2	0.0257	0.14
AMOT	Angiomotin	0.0335	0.14

Selection of DEG’s annotated to significantly enriched GO-terms associated to inflammation and cell death. The portrayed genes were selected due to possible biological significance for podocyte health. Adjusted *p*-value was considered significant at *p* < 0.05.

### MPA treatment influences terms related to inflammatory pathways and cell death

Another major group that came to our attention includes terms related to inflammatory pathways and cell death. Here, we could see an increase in terms describing the downregulation of inflammatory pathways, especially the p38-Mitogen activated protein kinase (p38MAPK) and the nuclear factor kappa-light-chain-enhancer of activated B cells (NF-κB) pathway. The *negative regulation of p38MAPK cascade* (FE 5.3, *p* = 0.001) presented itself as the GO-term with the largest fold enrichment (Fig. 1C). Notably, we found an increase in gene transcripts of the dual specificity protein phosphatase (DUSP) family, namely *DUSP1, DUSP4* and *DUSP6* (log2FC 0.23, 0.39, 0,31 and padj = 0.0004, 9.33E-05, 0.007 respectively). Treatment with MPA also increases the transcription of ubiquitin carboxyl-terminal hydrolase *CYLD* (log2FC 0.12, padj = 0.04), which is known to suppress the NF-κB, p38MAPK and c-Jun N-terminal kinase (JNK) cascades. Simultaneously, terms concerning the activation of these pathways through INF-χ and transforming growth factor-β (TGF-β) were significantly downregulated and therefore appeared in Cluster 1. One example of a downregulated transcript is *THBS1* (log2FC − 0.16, padj = 0.006), which, if translated, can lead to an activation of TNF-α and TGF-β pathways. The transcript with the largest negative fold change after MPA treatment is *PLK-1* (log2FC − 1.03, padj = 0.0004), which translates into a serine-threonine kinase with important functions in cell cycle regulation. In addition to the regulation of terms concerning inflammatory pathways, we could also see a surge of terms mentioning the negative regulation of cell death pathways, such as apoptosis and necroptosis. Mentionable examples of genes regulated by MPA and annotated to these functions are *CCN-I, PEA15A,* and *DDX3X* (Table 2).

**Table 2:** Genes associated to inflammation and cell death.

Name		Adjusted *p*-value	Log2-fold change
DUSP1	Dual specificityproteinphosphatase 1	0.0004	0.23
DUSP4	Dual specificityproteinphosphatase 4	9.33E-05	0.39
DUSP6	Dual specificityproteinphosphatase 6	0.0176	0.21
AK4	Adenylatekinase 4	0.0013	0.45
SLC25A4	ADP/ATP translocase 1	2.10E-10	0.32
CYLD	Ubiquitin carboxyl-terminal hydrolase CYLD	0.0442	0.12
TAX1BP1	Tax1-binding protein 1	0.0398	0.12
PLK-1	Serine/threonine-protein kinase PLK1	0.0004	− 1.03
CDK9	Cyclin-dependentkinase 9	0.0136	− 0.19
ARRB1	Beta-arrestin-1	0.0183	− 0.20
CXCL12	Stromalcell-derivedfactor 1	1.42E-06	− 0.26
LRP5	Low-density lipoprotein receptor-related protein 5	0.0346	− 0.17
THBS1	Thrombospondin-1	0.0056	− 0.6

Selection of DEG’s annotated to significantly enriched GO-terms associated to the actin cytoskeleton. The portrayed genes were selected due to possible biological significance for podocyte health. Adjusted *p*-value was considered significant at *p* < 0.05.

### MPA treatment is able to protect the actin cytoskeleton from BSA induced stress

To validate the biological significance of changes in the actin cytoskeleton related genes, we designed an experimental setup intended to damage actin filaments in a non-immunological manner and with resemblance to the pathophysiology of the nephrotic syndrome (Fig. 2A). We then stained the cells with Phalloidin, which interacts highly selectively with F-actin, to visualize the cytoskeletal structure of each cell (Fig. 2D). After 48 h of incubation with BSA*,* cells showed a disorganization of the actin cytoskeleton and an increased appearance of vacuoles. The quantification of the cytoskeleton revealed a substantial decrease of cells with thick cables (Type A and B) after treatment with BSA compared to controls. Simultaneously, the number of cells with thin or no cables (Type C and D) increased remarkably compared to control cells (Fig. 2B). Interestingly, the BSA + MPA group displayed a vastly different appearance, compared to the BSA + veh group. The number of Type A and B cells increased significantly compared to cells treated only with BSA (*p* = 0.004 and *p* = 0.001 respectively, Fig. 2B). Concomitantly, the share of non-stress fiber containing Type D cells decreased in a significant manner (*p* = 0.004, Fig. 2C), displaying an approximation of the actin cytoskeleton pattern of BSA + MPA cells, to that of control cells. The treatment with only MPA resulted in a similar distribution of cell types as in controls.

### MPA treatment reduces TNF-α and CHX induced cell death

Next, we examined consequences of the transcriptomic changes regarding inflammation and cell death in a functional environment. In view of the pathways that appear to be affected by MPA treatment, we chose TNF-α and CHX as appropriate, synergistically acting stressing agents, with a potent ability to induce cell death. The experimental design is shown in Fig. 3A. Using the neutral red uptake assay, we discovered that treatment with TNF-α and CHX reduced cell viability to only 36.8% (Fig. 3B). If simultaneously treated with MPA, no significant difference in cell survival was notable (data not shown). In contrast, cells with a treatment schedule including 24 h of pretreatment with MPA, showed a significantly reduced cell death rate, with 62.9% of cells viable after incubation with TNF-α and CHX (Fig. 3B, *p* = 0.008). Emricasan, an inhibitor of pan-caspases, was able to strongly diminish the cell damaging activity of the two agents, leaving 91% of cells viable after treatment. A proliferative effect due to MPA treatment was ruled out using an MPA-only group (Fig. 3B). To further validate our results and to elucidate the role of caspase-inhibition through MPA, we decided to perform a Western Blot analysis of CC3 levels. As expected, TC treatment strongly initiated Caspase-3 cleavage. More importantly, we found that additional MPA treatment decreased the amount of CC3 (Fig. 3C). A quantification of the western blot results can be found in the supplementary information (Supp. Fig. 2).

## Discussion

To our knowledge, we are the first to use an unbiased transcriptomic approach in order to investigate how MPA affects podocytes in a non-immunologic environment in a favorable manner. Interestingly, the result of the transcriptomic analysis showed that treatment with MPA for 24 h increased gene transcription of IMPDH II significantly (Fig. 1B), implying an effective exposure to MPA. This result is in line with past research, which had also demonstrated that murine podocytes express IMPDH transcripts and that IMPDH activity can be inhibited in cell culture^43^.

Hierarchical clustering identified 4 clusters, which were then analyzed for functional implications by conducting a GO-Term Enrichment Analysis. Upon closer examination we discovered that there was an accumulation of enriched terms associated to actin, especially in clusters of upregulated genes, such as *actin cytoskeleton* and *regulation of small GTPase mediated signal transduction* (Fig. 1C). Actin is the predominant cytoskeletal structure in FPs and as reviewed previously its reorganization or dysfunction can be a leading cause for proteinuria^44–46^. Since stabilization of the actin cytoskeleton has been demonstrated as a direct effect of cyclosporine A on podocytes^19^ we tested if MPA has a similar ability to directly act on the podocyte actin cytoskeleton. Therefore, we designed an experimental setup in which BSA was used as a stressing agent. In line with previous literature^37^, BSA treatment markedly decreased the amount and thickness of stress fibers. Notably, MPA was able to protect the cells from the albumin overload induced injury. Treatment with MPA reconstituted the number of actin rich Type A and B cells, while showing a significant decrease of Type D cells compared to the BSA + veh group (Fig. 2B,C).

This result strongly supported our concept of a stabilizing effect of MPA on the actin cytoskeleton. Next, we went back to our transcriptome data and looked at the actin cytoskeleton stabilization associated genes in more detail. The first interesting candidate was *SYNPO2L* (Table 1). *SYNPO2L* is a member of the synaptopodin family, which has been shown to stabilize the actin cytoskeleton in numerous studies^38,47–49^. If overexpressed *SYNPO2L* itself can activate the actin signaling pathway, increasing proteins like RhoA and Actn2 while inducing stress fiber formation^50^. Since synaptopodin is lost in childhood nephrotic syndrome^51^, an increase in a member of the synaptopodin family, like *SYNPO2L*, could potentially compensate for the loss of the actin supporting protein. Another interesting gene regulated through MPA is *ITGB1*, a major adhesion molecule in podocytes, which is downregulated under mechanical stress as well as under albumin overload, both being leading pathological mechanisms in INS^52,53^. The decrease of *ITGB1* can result in a reduced podocyte adhesion and an increase in Caspase-3 activity^54^. Lee et al. were able to show that pyrintegrin, a beta1-integrin-agonist, is able to protect murine podocytes from effacement and subsequently mice from proteinuria^55^. In our study we discovered that MPA increases ITGB1 gene transcription, thereby possibly making use of its stabilizing effect on podocytes.

Further examination of the RNAseq-data revealed another group of terms, including negative *regulation of p38MAPK* and negative *regulation of apoptotic process* in the upregulated clusters and a negative *regulation of TGF-*β *receptor signaling pathway*, that are linked to cell death and inflammation (Fig. 1C). The p38MAPK-pathway is a well-known pro-inflammatory and pro-apoptotic pathway, that is among other things activated during endoplasmic reticulum stress (ERS)^56,57^. ERS, TGF-β and p38MAPK have all been shown to be upregulated through albumin overload in podocytes in vitro^37,58^. Koshikawa et al. were able to demonstrate that p38MAPK-activation is increased in rodent kidney disease models, as well as in human glomerulopathies, including MCGN and FSGS^59^. In line with these findings, the inhibition of TGF-β and p38MAPK proved to be protective for podocytes in vitro and in vivo^37,59,60^. Considering our transcriptomic analysis together with the aforementioned studies, we tested if MPA has the ability to protect podocytes from inflammation and subsequent cell death and examined this hypothesis using a cell viability assay. Previous work had shown that nephrotic syndrome in mice and albumin exposure to podocytes in vitro increase TNF-RNA^61^, which in turn can induce podocyte injury and glomerular disease progression^62,63^. We therefore treated our cells with a routinely used combination of TNF-α and CHX, which showed a massively detrimental impact on podocyte survival. Nonetheless we were able to demonstrate a significantly higher survival of cells pre-treated with MPA compared to those not treated with MPA (62.9% vs. 36.8%, Fig. 3B). Thus, our data impressively demonstrate that TNF-α -induced cell death can effectively be mitigated when cells are pretreated with MPA. On the contrary, treatment with MPA after stimulation with TNF-α was not successful in preventing cell death. Subsequently, we demonstrated that MPA treatment reduces Caspase-3 cleavage induced by TC treatment (Fig. 3C). Taken together, we were able to show that MPA attenuates TNF-α induced cell death in cultured podocytes. Mechanistically, this can at least partially be related to a reduction of Caspase-3 activation.

Strikingly, our transcriptomic results showed the upregulation of three members of the DUSP family after MPA treatment, namely *DUSP1*, *4* and *6*, which are negative regulators of MAPK-pathways including the p38- and JNK-pathway (Table 2)^64^. *DUSP4* and *6* have both been found to be decreased in diabetical nephropathy models^65,66^. If overexpressed in podocyte culture, *DUSP4* decreases the activation of p38MAPK, JNK and Caspase-3^65^. Similarly, overexpression of *DUSP6* protects podocytes from synaptopodin and nephrin loss, while reducing high-glucose induced apoptosis^66^.

Other mentionable genes affected by MPA that could potentially play a role in the treatment of proteinuric diseases include *PLK-1* and *CXCL12*, which were both downregulated through MPA treatment (Table 2). *PLK-1* is a key regulator of the cell cycle and cell division and its inhibition has been shown to reduce proteinuria in a diabetic nephropathy model^67,68^. *CXCL12*, also known as stromal cell derived factor, is a homeostatic chemokine, whose production by podocytes has been shown to contribute to podocyte loss and albuminuria in type 2 diabetes^69^. Its blockage ameliorates proteinuria and increases podocyte numbers in models of both diabetical nephropathy and adriamycin-induced nephropathy^70,71^.

Taken together, MPA does indeed protect the actin cytoskeleton and can prevent the cell death of podocytes in cell culture. Although our results have the usual limitations of cultured podocytes regarding cell type specificity and environmental surrounding, the cell culture model is highly valuable to study effects beyond those of immune cells on podocytes, which are confounding the results in almost all in vivo models. Nevertheless, future studies will have to examine the translatability of our findings into animal models, as well as compare our findings to transcriptomic analysis of human samples. Understanding the mechanisms of action of clinically used immunosuppressive drugs in INS will help to further adapt therapeutic regimes to fully exploit its therapeutic potential. In conclusion, this study provides compelling evidence for a direct favorable effect of MPA on podocytes, while providing potential responsible pathways.

## Supplementary Information

Supplementary Information 1.

Supplementary Information 2.

Supplementary Information 3.

Supplementary Information 4.

Supplementary Information 5.

## Data Availability

Sequencing data are available in the ArrayExpress database under the following link: https://www.ebi.ac.uk/biostudies/arrayexpress/studies/E-MTAB-11702?key=76ebcb9d-12e4-4e3b-b0e2-cada7edc4bf5.
