# Supplementary material for: Mycophenolic acid directly protects podocytes by preserving the actin cytoskeleton and increasing cell survival
Source: Sci Rep. 2023 Mar 15;13:4281. doi: 10.1038/s41598-023-31326-z (PMC10017704; doi:10.1038/s41598-023-31326-z)

Mycophenolic acid directly protects podocytes by preserving the actin cytoskeleton and increasing cell survival

⊥ Seif El Din Abo Zed [1,2]; *⊥ Agnes Hackl MD PhD [1,2]; Katrin Bohl PhD [2,5]; Lena Ebert PhD [2,5]; Emilia Kieckhöfer [2,5]; Carsten Müller MD [3]; Kerstin Becker PhD [4;] Gregor Fink PhD [1]; Kai-Dietrich Nüsken MD [1]; Eva Nüsken MD [1]; Roman-Ulrich Müller MD [2,5,6]; Bernhard Schermer MD [2,5]; Lutz T. Weber MD [1]

[1]Faculty of Medicine and University Hospital Cologne, University of Cologne, Department of Pediatrics, Cologne, Germany

[2]Faculty of Medicine and University Hospital Cologne, University of Cologne, Department II of Internal Medicine and Center for Molecular Medicine Cologne, Cologne, Germany

[3]Faculty of Medicine and University Hospital Cologne, University of Cologne, Pharmacology at the Laboratory Centre, Department of Therapeutic Drug Monitoring DE, Cologne, Germany

[4]Faculty of Medicine and University Hospital Cologne, University of Cologne, Cologne Center for Genomics (CCG), Cologne, Germany

[5]University of Cologne, Cologne Cluster of Excellence on Cellular Stress Responses in Ageing-Associated Diseases (CECAD), Cologne, Germany

[6]Faculty of Medicine and University Hospital Cologne, University of Cologne, Center for Rare Kidney Diseases Cologne, Cologne, Germany

⊥ S. Abo Zed and A. Hackl contributed equally to this work

*Corresponding author:

Dr. Agnes Hackl

Faculty of Medicine and University Hospital Cologne, University of Cologne, Department of Pediatrics,

Kerpener Strasse 62, 50937 Cologne, Germany

Fax number: +49 221 478 1431763

Telephone number: +49 221 478-42101

E-Mail address: agnes.hackl@uk-koeln.de

Supplementary Figure 1 Uncropped images of western blot results regarding cell viability assay. (A) Western Blot Analysis of full-length Caspase-3 (Casp-3 Fl) and cleaved Caspase-3 (cleaved Casp-3) levels. **(B)** Western Blot analysis of housekeeping proteins pan-actin and Gapdh.

A

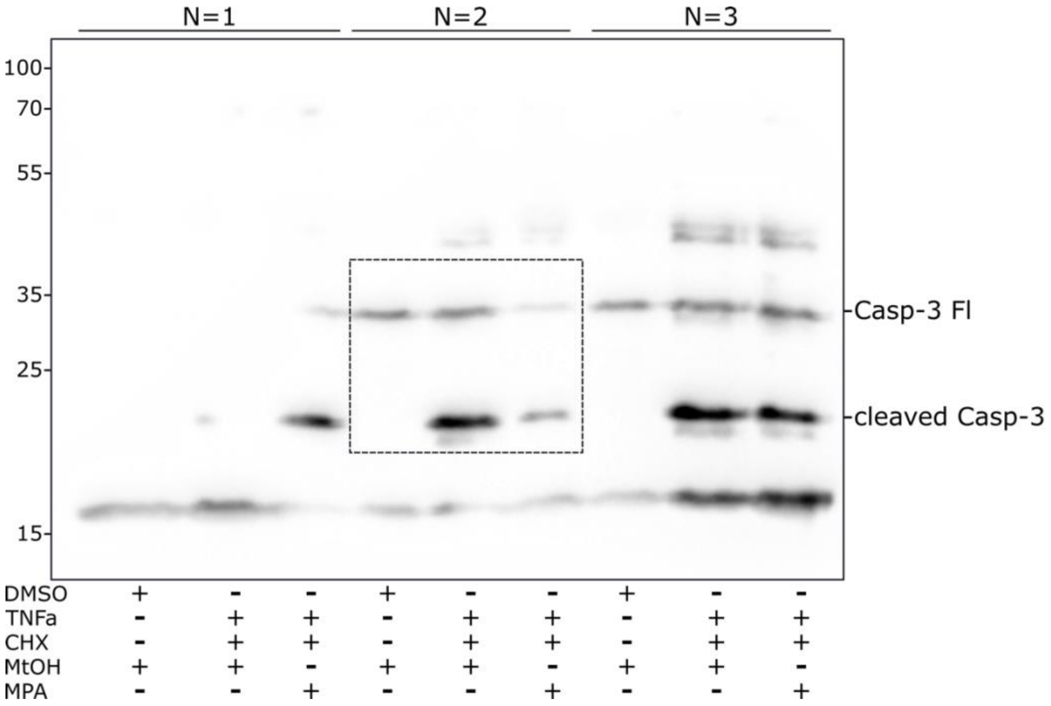

B

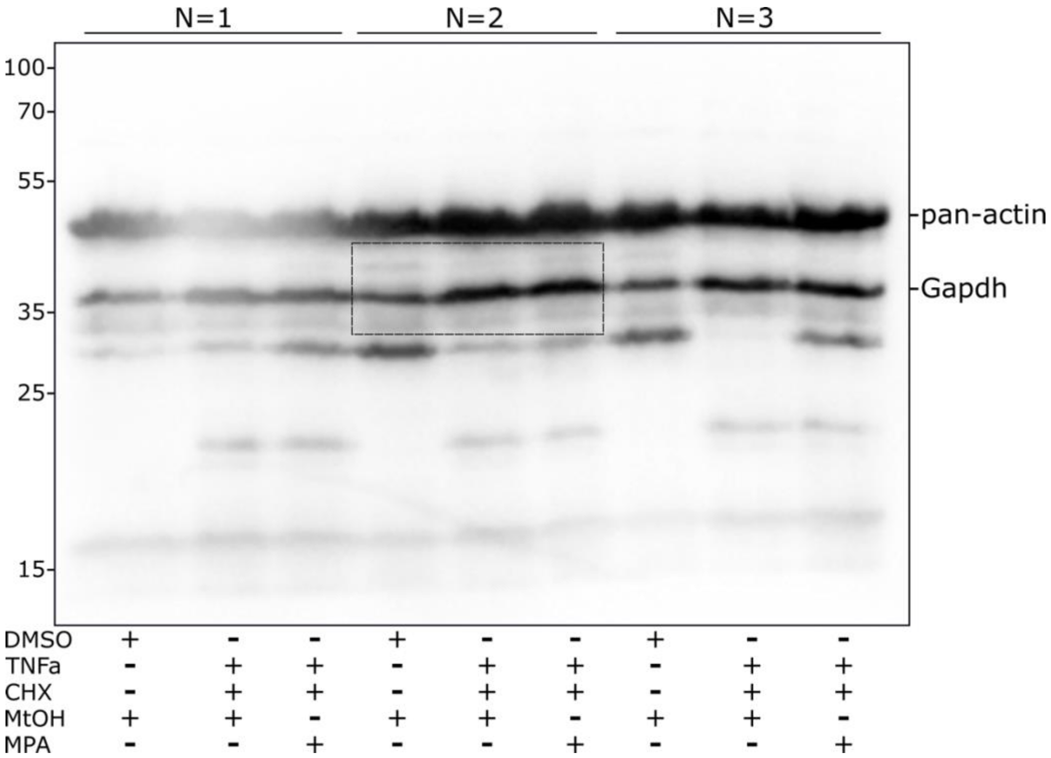

Supplementary Figure 2 Quantitative analysis of relative cleaved Caspase-3 expression normalized to Gapdh. Cells were treated with combinations of Tumor Necrosis Factor-α (T), Cycloheximide (C) and Mycophenolic Acid (M). Pretreatment with MPA shows a reduction of relative cleaved Caspase-3 expression compared to cells treated only with TC. *$p<0.05$

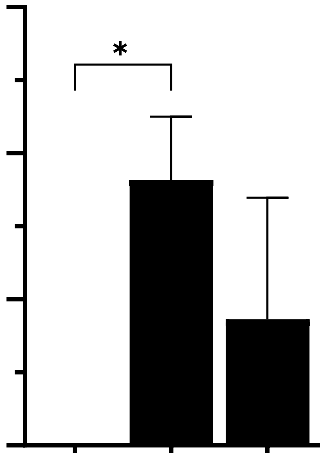

Supplement: Supplementary file 5 — Supplementary Information 5. [file 41598_2023_31326_MOESM5_ESM.pdf]
